# Using Ambient Scent to Enhance Well-Being in the Multisensory Built Environment

**DOI:** 10.3389/fpsyg.2020.598859

**Published:** 2020-11-19

**Authors:** Charles Spence

**Affiliations:** Department of Experimental Psychology, Crossmodal Research Laboratory, University of Oxford, Oxford, United Kingdom

**Keywords:** smell, scent, built environment, multisensory, malodor, well-being, sensehacking

## Abstract

The majority of the world’s population now lives an urban existence, spending as much as 95% of their lives indoors. The olfactory atmosphere in the built environment has been shown to exert a profound, if often unrecognized, influence over our mood and well-being. While the traditionally malodorous stench to be found indoors (i.e., prior to the invention of modern sanitation) has largely been eliminated in recent centuries, many of the outbreaks of sick-building syndrome that have been reported over the last half century have been linked to the presence of a strange smell in the environment. At the same time, however, there is also growing evidence that consumer behavior can be manipulated by the presence of pleasant ambient odors, while various aromatherapy scents are said to improve our mood and well-being. This Anglophone review focuses primarily on indoor western urban developed spaces. Importantly, the olfactory ambience constitutes but one component of the multisensory atmosphere and ambient odors interact with the visual, auditory, and haptic aspects of the built environment. Surprisingly, the majority of published studies that have deliberately chosen to combine ambient scent with other sensory interventions, such as, for example, music, have failed to increase store sales, or to enhance people’s mood and/or well-being, as might have been expected. Such negative findings therefore stress the importance of considering multisensory congruency while, at the same time, also highlighting the potential dangers that may be associated with sensory overload when thinking about the effect of ambient smell on our well-being.

## Introduction

The proportion of the world’s population living an urban existence continues to grow year-on-year. In fact, as of 2010, more people around the globe lived in cities than in rural areas, and by 2050, it has been estimated that 60% of the world’s population will be urban (see UN-Habitat, 2010; United Nations Department of Economic and Social Affairs, 2018). Given that city dwellers spend something like 90–95% of their lives indoors (Ott and Roberts, 1998; Klepeis et al., 2001; Wargocki, 2001; Velux YouGov Report, 2018), the diet of multisensory stimulation that the majority of us are exposed to on a daily basis is likely very different and, most probably, much more monotonous (see Draycott, 2015, p. 60), than that we may have once evolved to deal with. Indeed, of America, Hall (1966, p. 45) once complained that the *“extensive use of deodorants and suppression of odors in public spaces results in an olfactory blandness and sameness that would be difficult to duplicate anywhere else in the world.”* Louv (2005), meanwhile, has written of ‘Nature Deficit Disorder.’ Nevertheless, a growing body of empirical evidence now shows that the ambient smells of the built environment can still have a profound effect on us, no matter whether we are aware of them or not (see Sobel et al., 1999; Hummel et al., 2006), and very often the evidence suggests that we are not. While unpleasant, and often unidentified, odors can undoubtedly have a negative impact, a range of pleasant scents have also been shown to exert a positive effect on people’s mood, their well-being, and their behavior/performance across a wide range of everyday situations (Spence, 2002, 2020e, 2021).

One of the problems, though, with olfactory perception is that we are visually dominant (see Hutmacher, 2019, for a recent review), and hence tend to give little consideration to the ambient smells that surround us (Jiang et al., 2016). Our tendency to focus our attention on what we see and hear means that we can often exhibit ‘olfactory anosmia’ to the presence of ambient scents (Forster and Spence, 2018; see also Sela and Sobel, 2010; and Spence, 2019, for a review). We also adapt rapidly to constant ambient odors, such as the smell of our own homes, only becoming aware of the distinctive building odor (or BO; McCooey, 2008) ourselves, as others presumably normally experience it, when, for instance, returning after a long trip away (Dalton and Wysocki, 1996; though see also Hummel et al., 2004). Here, though, it should be noted that while we adapt to ambient odors just as long as they are pleasant or neutral. We typically fail to adapt to constant unpleasant ambient odors though (Matheny and Honoré, 2011; Anon, 2018). We are very suggestible as far as the presence and/or pleasantness of ambient odors are concerned. For instance, Slosson (1899) conducted a now-classic demonstration in which the students in a university lecture theater were instructed to raise their hands on detecting an odor in the air that the professor had apparently released from a bottle at the front of the auditorium. Slosson (1899) reports how a slow wave of raised hands could be seen moving from the from to the back of the lecture theater, despite the fact that the professor had not actually released any scent at all (see also Knasko et al., 1990; van den Bergh et al., 2004).

### The Ambient Smell of the Multisensory Environment

As we will see later, one cannot simply focus solely on the olfactory ambience, and ignore the visual, auditory, and even tactile aspects of the built environment. This is because the senses combine to influence our overall experience of, and response to, the multisensory atmosphere of the spaces in which we live, work, play, and sleep (Spence, 2002, 2021). And while a number of architectural theorists have, in recent decades, increasingly started to stress the importance of going beyond the purely visual aspects of design (e.g., Anderton, 1991; Pallasmaa, 1994, 1996, 2000; McCarthy, 1996; Malnar and Vodvarka, 2004; Eberhard, 2007; see also de Vries, 1997), they have not, as yet, fully recognized the multisensory interactions that drive our holistic response to the built environments in which we spend so much of our lives (see Spence, 2020e, for a recent review).

At the same time, however, a growing body of cognitive neuroscience research has revealed the various rules the brain uses in order to combine sensory inputs, sometimes doing so in what may at first appear to be surprising ways (Spence, 2021). Sometimes, for example, one sense dominates over the others in terms of determining our behavioral/perceptual response. Typically, vision tends to be the dominant sense (Hutmacher, 2019, for a review). At other times, though, weak unisensory cues (such as faint scents and sounds) can combine to give rise to a multisensory response that appears to be bigger than the sum of the parts (see Stein and Meredith, 1993; Lwin et al., 2010). However, should the senses provide conflicting, or incongruent, information, then the resulting gestalt can be hard for people to process, thus leading to a loss of processing fluency that is normally negatively valenced (e.g., Reber et al., 1998, 2004; Winkielman et al., 2003, 2015; Reber, 2012; Herrmann et al., 2013).

## Eliminating Malodor

It is widely accepted that the built environment would traditionally have smelled terrible (e.g., Bradley, 2015). Indeed, it has long been suggested that prior to the widespread introduction of flushing toilets (invented at the end of the 16th century, but only in widespread use in the latter-half of the 19th century; Stamp, 2014), there would have been unpleasant odors both within and without the home (e.g., Jütte, 2005; see Engen, 1982, pp. 135–136; and Corbett, 2006, on the universal Western dislike of toilet smells). Or, as Potter (1999, p. 169) once succinctly put it: *“There can be no question but that the urban air of the Roman empire stank.”* The interesting thing to note, though, is how those living under what were presumably extremely smelly conditions (e.g., in Roman times) never appeared to comment on the stench, instead choosing to complain about the goaty/garlicky smell of those living in the countryside instead (Morley, 2015, p. 117). That said, the richest residents of Ancient Rome were known to pride themselves on spraying their toilets with perfume, and, on notable occasions, releasing perfumed doves to try and scent the air while feasting/dining, suggesting that they were at least aware that the normal smell of their homes was undesirable (see Bradley, 2015; Koloski-Ostrow, 2015).

Moving forward in time, Corbin (1986) has also highlighted the deodorization of everyday life that occurred in France during the 18th century (see also Payer, 1997; Jenner, 2000, 2011; Jütte, 2005, pp. 207–211; el-Khoury, 2006; Cockayne, 2007; Chiang, 2008, for a description of the similar changes that were taking place elsewhere in Europe and North America). Here, I should stress that this Anglophone review will focus primarily on indoor western urban developed spaces, as this is where the majority of the literature can be found. There has long been a belief in the connection between unhealthy foul odors and pestilence/disease (Jenner, 2011), with many people using nosegays and other fragrant handheld objects (e.g., pomanders) in order to try and help mask unpleasant environmental smells (Classen et al., 1994; Brant, 2004). Early reports of the use of nosegays can be traced back at least as far as Ancient Roman times (Morley, 2015, p. 117). Meanwhile, according to Corbett (2006, p. 224), *“Regulation of air flows was crucial to Enlightenment architects. Buildings were designed so as to separate putrid exhalations from currents of fresh air, in the same way that fresh water had to be divided from used water. The degree of stench became the measure of the architect’s efficiency.”* Improvements in sanitation and personal hygiene gradually helped to reduce the malodor in city streets as well as in the home. Furthermore, the use of perfume also became cheaper and hence more widespread in Europe during the 19th century (see Jenner, 2011, p. 340).

Complaints and concerns about the malodor of the masses continued, especially in those locations where the great unwashed would sometimes congregate. So, for example, moving into the opening decades of the 20th century, there were various attempts to deal with the sweaty stench of unwashed bodies crammed together in the poorly ventilated early cinemas (see Spence, 2020d, for a review). In this case, the smell became so unbearable that regular 10 min airing breaks were introduced (Payer, 2001). On occasion, deodorant (Perolin, which had a naphthalene-like smell) was even sprayed out over the audience (Berg-Ganschow and Jacobsen, 1987). The widespread introduction of air-conditioning in cinemas in the 1930s would, though, presumably have helped address the problem of bodily malodor in warmer climates (see Arce, 1979, p. 104; Heschong, 1979).

Similar problems with malodor were likely to have affected early music hall, vaudeville, and possibly also theater/opera venues as well. Fragranced fountains were, on occasion, introduced into the lobbies of London theaters in the latter decades of the 19th century (Rimmel, 1865; Anon, 2020), presumably to help mask the smell of the audience. The underlying problem of malodor may not have been as bad in the theater as it was in the cinema, where multiple daily screenings would also have been the norm. What is more, cinemagoers were more likely to go straight from work, whereas those going to the single theater performance of the day would have been more likely to get changed (and possibly also washed) beforehand. Adding to the disparity between the cinema and theater at the start of the 20th century, one German engineer calculated that the latter had as much as three or four times more air to breathe per person (Richter, 1926).

Intriguingly, researchers in Germany recently assessed the composition of the ‘audience emitted chemicals,’ comprising various volatile organic compounds (VOCs), such as carbon dioxide and isoprene (C_5_H_8_), from cinemagoers’ sweat and breathing, while watching a range of movies in a 250-seater cinema in Mainz (Williams et al., 2016). Data were collected from more than 9,500 people while watching one of 108 screenings of 16 different films. Changes in the chemical composition of the atmosphere given off by the audience could be linked to the on-screen action, with a significant increase in chemicals being associated with thrill and comedy scenes. Such results therefore hint at the possibility that volatile human chemosignals from the rest of the audience might actually contribute to the viewer’s multisensory experience. The authors themselves go on to suggest that: “the chemical accompaniment generated by the audience has the potential to alter the viewer’s perception of a film” (Williams et al., 2016, p. 7; see also de Groot et al., 2015). Rather more worryingly, though, thirdhand exposure to smoke has also been documented in cinemagoers when those who have been smoking enter the auditorium (Sheu et al., 2020).

Moving forward to the end of the 1930s, concern about the detrimental effects of so-called ‘store odor’ (SO) started to appear in trade publications directed at the owners of North American food stores (Anderson, 1939; see Mack, 2010, for a review). In this case, the smells that store managers were told to avoid were those associated with stale, rotten, or spoiled produce. Fresh smelling and deodorized is what the store owners were told to aim for instead (Cline, 1941). More recently, following the widespread ban on smoking, many venues, such as nightclubs, started to smell rather unpleasant – or rather, the unpleasant stale odor was no longer masked by the smoke (see Schifferstein et al., 2011). Recognizing the widespread problem of malodor in such enclosed spaces, Schifferstein et al. (2011) reported how the dance club experience could be enhanced simply by introducing a pleasant scent. They reported that the scents of orange, seawater, and peppermint were all equally efficacious, compared to a no scent baseline, in terms of enhancing dancing activity, people’s evaluation of both the music and the evening, and the latter’s mood (based on almost 850 completed questionnaires). Interesting in this regard, according to press reports (see White, 2011), the China White nightclub in London started using ambient scent to improve the ambience at around the same time. Four years earlier, in 2007, there were also press reports circulating that the Luminar chain of nightclubs and venues was pumping a rose scent through the air-conditioning to counteract the stale smell of sweat and beer (Anon, 2007).

While the problem of malodor in the built environment has seemingly been largely eliminated in the modern era (though see Schiffman et al., 1995; Rinck et al., 2011), one nevertheless still comes across occasional reports of problematic ambient smells. For example, a citrus scent was introduced onto the underground carriages in Vienna recently in order to address growing complaints concerning the sweaty malodor associated with passengers traveling during the summertime (Walker, 2019). It is unclear whether the deliberate scenting of other public spaces, such as the much commented on Barclays Center arena in Brooklyn, NY, United States, home of the Brooklyn Nets, on its opening in 2013, might not also have been introduced in order to cover up the sweaty smell of the fans (Albrecht, 2013; Doll, 2013; Martinez, 2013). At the same time, however, it is worth noting that problems have sometimes resulted from the synthetic scenting of enclosed spaces, such as the underground in the United Kingdom (Jury, 2002).

On the rare occasions nowadays where malodor intrudes into our olfactory experience in the built environment, it can exert a significant impact on both our well-being and behavior. For example, writing in The New York Times, Pacelle (1992) noted how many people refuse to check in, if they detect a strange smell in the hotel lobby. The growing awareness of such olfactory problems presumably helping to explain the rise of signature scents that are nowadays often to be experienced in the lobbies of many hotel chains (Goodwin, 2006; Stellin, 2007; Kaufman, 2017). Meanwhile, in 2016, Virgin Australia had a problem when some of its passengers started falling sick at what they interpreted as the smell of sweaty socks, but which was actually the parmesan cheese sandwiches that they had started selling (Buaya, 2016). Note that the same chemical isovaleric acid is found in both, hence explaining why this is such a popular compound for research into the cognitive modulation of olfactory perception (e.g., Herz and von Clef, 2001; De Araujo et al., 2005). Laboratory research by Rotton (1983) has demonstrated the affective and cognitive impact of ambient malodor released into a testing room (see also Rotton et al., 1978; Weber and Heuberger, 2008, Experiment 5).

### Interim Summary

While efforts to deodorize, or else mask, unpleasant ambient odors in the built environment have been largely successful (Stenslund, 2015), the ensuing total absence of smell in many contemporary environments, such as the art gallery, and other cultural institutions, has led some commentators to complain. For example, Drobnick (2005) has criticized the ‘anosmic cube’^1^ mentality that has taken hold in many public spaces (see also Byatt, 2003). Oftentimes, it would appear that it is people’s beliefs about the environmental source of an ambient odor that is key to determining their responses, rather than necessarily the nature of the physical stimulus itself (Dalton, 1996).

While the built environment was likely much smellier in former times than it is today, people may simply have either adapted, or else habituated, to the ever-present malodor, meaning that it may not have smelled as bad to them as it would to us today if we were exposed to the same stench. As Engen (1982, p. 169) notes: *“Odor preferences are acquired by learning to adapt to the environment.”* What is more, he also argues that people would once presumably have habituated to what now we would consider a terrible smell (see Engen, 1982, pp. 63–77).

## Sick-Building Syndrome

Sick-building syndrome (SBS) is the name given to illness caused by air pollution in the built environment (Rothman and Weintraub, 1995; Joshi, 2008; Sahlberg, 2012), possibly caused by the build-up of VOCs (Love, 2018). According to Joshi, the symptoms include: Headache, dizziness, nausea, eye, nose or throat irritation, dry cough, dry or itching skin, difficulty in concentrating, fatigue, enhanced sensitivity to odors, hoarseness of voice, allergies, cold, flu-like symptoms, increased incidence of asthma attacks and even personality changes (see also Anon, 2000). In the context of the present special issue (*Smell, Well-Being, and the Built Environment*), it is interesting to note how many of the large outbreaks of SBS in the 1980s were linked to the presence of an unfamiliar smell in closed office buildings with little natural ventilation (see also Wargocki et al., 2000).

For instance, back in June 1986, more that 12% of the workforce of 2,500 people working at the Harry S. Truman State Office Building in Missouri came down with the symptoms of SBS over a 3-day period (Donnell et al., 1989). The symptoms presented by a number of the office workers, including headaches, mucosal irritation, fatigue, dizziness and an odd taste, were so severe they had to be taken to the local hospital for emergency treatment. While a thorough examination of the building itself subsequently failed to uncover any particular toxic airborne pollutants that might have triggered the outbreak, the symptoms of SBS were, in the majority of cases, preceded by the perception of unusual odors and inadequate airflow in the building. It has also been suggested that the energy crisis in the 1970s may also have been partly to blame, as that tended to result in lower ventilation standards. According to Donnell et al. (1989), these complaints regarding the presence of a strange odors may well have heightened the building employees’ perception of poor air quality. This, in turn, may then have led to an epidemic anxiety state (mass hysteria) resulting in the outbreak itself (Faust and Brilliant, 1981; Rothman and Weintraub, 1995). Those workers suffering from SBS were more than twice as likely to have noticed a particular odor in the office before the onset of their symptoms than those who were unaffected by the outbreak.

To give a sense of the potential scale of the problem, Woods (1989) estimated that 30–70 million people in the United States alone are exposed to offices that manifest SBS. As such, anything (and everything) that can be done to reduce the symptoms associated with this reaction to the indoor environment will likely have a beneficial effect on the health and well-being of many office workers (Finnegan et al., 1984). Those who believe that it is the neglect of the olfactory aspects of the interior environments that may be at least partly responsibly for SBS, have argued that improving indoor air quality might therefore provide an effective means of helping to alleviate the symptoms (e.g., Guieysse et al., 2008). Consistent with such a view, researchers have demonstrated that both increased ventilation rates, and reduced air pollution, can exert a significant influence over the subjective well-being, SBS symptoms, and performance (e.g., typing speed) of female office workers (Wargocki et al., 1999, 2000).

At the same time, however, it is perhaps also worth bearing in mind here that reports of SBS in the office environment would appear to have declined somewhat in recent decades, perhaps suggesting (at least according to certain commentators) that commercial building design/ventilation has improved as a result of the earlier outbreaks. The indoor smoking bans introduced in many countries are also likely to have resulted in improved indoor air quality (though see Sheu et al., 2020). A more pessimistic take, though, would instead be to suggest that many of the symptoms of SBS (which is not, it should be noted, a recognized illness; Anon, 2000) are now so ubiquitous in society today that they no longer merit any special mention (see also Magnavita, 2015; Higham, 2019; Kim et al., 2019).

Over the years, there have been a number of reports suggesting that indoor plants might provide an effective means of helping to absorb the VOCs (Wolkoff et al., 2006) that tend to build up indoors (e.g., Brown et al., 1994; Guieysse et al., 2008), as well as possibly providing a range of other psychological benefits to the occupants of office buildings (Bringslimark et al., 2009; Nieuwenhuis et al., 2014). However, the latest research suggests that the number of plants that would be needed to exert a significant effect on VOCs is realistically too large for all but the Amazon HQs of this world to achieve (Walker, 2000; Cummings and Waring, 2020). Amazon HQ, known as The Spheres, and situated in downtown Seattle, consists of a biome of three huge plant-filled domes (Hartmans, 2018), housing an estimated 40,000 plants, encompassing more than 400 different species (see also Everett, 2019).

At the same time, however, there continue to be many reports of Sick Home Syndrome (SHS), especially amongst those living in cooler Northern climates, such as in Scandinavian countries (e.g., Finland; Runeson-Broberg and Norbäck, 2013; Love, 2018). In the latter case, it is the damp/mold in the home environment that is thought to be responsible. That said, there continues to be some uncertainty as to whether the symptoms of SBS should be attributed to airborne pollutants, or may instead be better understood as a psychosomatic response to a particular environmental atmosphere (see Redlich et al., 1997; Shusterman, 2001; Fletcher, 2005; Love, 2018). Indeed, one of the psychological factors that has been suggested to be relevant here concerns the feeling of a lack of control over one’s multisensory environment that many of those working in ventilated buildings experience, especially when the windows cannot be opened manually (Faust and Brilliant, 1981).

A number of researchers now prefer to use the less pejorative term building-related symptoms (BRS) instead of SBS (Niemelä et al., 2006). At the same time, however, it should be recognized that some people also suffer from multiple chemical sensitivity (MCS), otherwise known as idiopathic environmental intolerance (IEI) that may well be triggered by the presence of ambient volatiles chemicals (Shusterman, 1992; IPCS, 1996; Van den Bergh et al., 2001; Bornschein et al., 2002; Fletcher, 2005). Though, as Fletcher (2005, p. 380) notes, while the majority of adverse reactions do indeed appear to be triggered by the presence of ambient smells, some episodes may also be triggered by foods and/or by physical contact with synthetic materials as well (see also Dager et al., 1987). MCS is characterized by the presence of a wide range of non-specific symptoms, such as fatigue and weakness, cognitive difficulties (concentration and memory), dizziness, pounding heart, shortness of breath, anxiety, headache, and muscle tension (notice here the overlap with the symptoms of SBS), in response to the presence of (often odorous) chemical substances present at concentrations below those known to cause harmful effects in the population.

### Interim Summary

Sick-building syndrome and SHS (not to mention BRS, MCS, and IEI) are amongst the most noticeable negative influences of poor indoor air quality on our well-being. Several of these conditions are often preceded by the inhabitants’ awareness of a strange smell in the environment. While the levels of certain carcinogenic chemicals in buildings may well be higher than is desirable (Nazaroff and Weschler, 2004), more often than not the deleterious effects of ambient smell on people’s well-being tend to result from a process of associative learning with an arbitrary odorant. Indeed, associative learning has often been documented for olfactory stimuli, whereby an ambient smell takes on negative associations (Herz, 2007), possibly due to unconscious odor conditioning (Kirk-Smith et al., 1983; though see Black and Smith, 1994; Epple and Herz, 1999; Zucco et al., 2009). This is where a scent can be conditioned (after being paired with an emotionally meaningful task, say) through exposure to be either positive or negative, despite the fact that the person is unaware of the odor having been presented. Subsequent exposure to the odor can then elicit a conditioned effect on a person’s mood. Evaluative olfactory conditioning is, by now, a well-studied phenomenon (Engen, 1988; Zucco, 2012). The negative consequences of exposure to certain ambient volatile stimuli that has been linked to cases of MCS is also thought to reflect learning (i.e., Pavlovian conditioning; Van den Bergh et al., 2001). It is precisely this kind of odor conditioning that has led some to suggest that workers should make sure to change the ambient odor after a stressful meeting in order to try and avoid such negative ambient smell-related effects (Spence, 2002). In some extreme cases, specific odors have even been shown to trigger post-traumatic stress disorder (e.g., Kline and Rausch, 1985; Vermetten and Bremner, 2003).

## Stressful Smells

The associative nature of the emotions that are attached by people to scent means that those smells that we tend to link to stressful/unpleasant healthcare situations, such as the eugenol (i.e., clove) smell of a visit to the dentist’s surgery, can all too easily end-up making us stressed (Robin et al., 1998, 1999). Back in the 1960s, one commentator was already recommending that hospitals experiment with ‘odor therapy’ by blowing pleasant scents into the wards so as to promote feelings of security and well-being amongst the patients (Hamilton, 1966; Stenslund, 2015, for a contemporary take on hospitals and ambient smell). There has also been a suggestion that certain ambient scents can also facilitate wound healing (Busse et al., 2014).

The research shows that simply changing the ambient aroma (e.g., replacing the clove smell with a citrus scent instead), can sometimes help to reduce the physiological signs of stress (Lehrner et al., 2000, 2005). Note, though, that not everyone has found the smell of citrus (or apple) to reduce anticipatory stress in patients waiting for treatment in the dental surgery (Toet et al., 2010). Here, though, it is worth noting that the citrus scent would itself eventually presumably also come to elicit stress should that become the standard smell that a person associates with a trip to the dentist. As such, it might be argued that healthcare establishments really ought to change their scent on a regular basis, say once every year or so, to avoid too many of their customers from building up an undesirable association between the stress of a visit and a particular ambient smell (Spence, 2021). At the same time, however, such a dynamic approach to scenting the healthcare environment does not leave much scope for establishing a healthcare brand’s signature scent, this is something that is becoming increasingly important to private healthcare providers working in what has become an increasingly competitive marketplace in a number of countries (see Goldkuhl and Styvén, 2007; Naja et al., 2014).

Over the years, researchers have also investigated the use of fragrance to help reduce stress during other anxiety-inducing medical procedures (e.g., Graham et al., 2003; Braden et al., 2009; Redd et al., 2009; Kritsidima et al., 2010; Ghiasi et al., 2019). There is also a growing body of research to suggest that ‘sweet-smelling’ ambient scents can help people deal a little better with pain (e.g., Prescott and Wilkie, 2007; though see also Marchand and Arsenault, 2002; Martin, 2006).^2^ Researchers have also demonstrated how the pleasantness of interpersonal touch can be modulated by the presence of either a pleasant or disgusting ambient scent (e.g., Croy et al., 2015, 2016).

Perhaps hinting at the potential for well-being interventions in this area, at least one patent has been awarded relating to the stress-relieving properties of certain essential oils either when inhaled or else applied transdermally (Warren et al., 1987). Summarizing almost a decade of research on the mood benefits of fragrance for IFF (the latter, one of the world’s largest fragrance houses), Warren and Warrenburg (1993, p. 9) arrived at the following surprisingly modest conclusions: *“1. Fragrance-evoked mood changes are small, but beneficial to our well-being. 2. Fragrance can be used to reduce the stress response in humans, but its physiological effects on a non-stressed subject are minimal and difficult to measure. 3. Measurement of fragrance-evoked mood change by psychological methods is feasible, and yields intriguing results.”* Warren and Warrenburg ended-up concluding their review by suggesting that: *“We believe that further improvements in our measurement techniques, and a better understanding of the mood changes evoked by specific perfumery ingredients will allow for the development of more impactful mood-altering fragrances”* (Warren and Warrenburg, 1993, p. 16).

## Using Ambient Scent to Enhance Well-Being

It has long been recognized that ambient odors can influence our well-being (e.g., Montaigne, 1993; Hosey, 2013). While, as we have just seen, undesirable, and negatively valenced, malodors tend to be associated with negative outcomes on the health, well-being, and mood of those who are exposed to them, it is important to note that pleasant ambient scents can also be used to help improve our mood and well-being (e.g., Spence, 2003; Glass et al., 2014; Glass and Heuberger, 2016; Haehner et al., 2017). Here, one can think of everything from the use of aromatherapy scents (see Spence, 2003; Herz, 2009; Perry and Perry, 2018, for reviews) through to the recent trend to deliver well-being flower bouquets (Carlyle, 2020). The latter supposedly not only looking beautiful but also exerting a positive influence over the olfactory environment. Scented candles have also become increasingly popular (Rose, 2019), though they may not be without their own risks in terms of the release of pollutants into the atmosphere (e.g., Lau et al., 1997; Lambert, 2012; though see also Petry et al., 2014, for the suggestion that concerns may, in fact, be unwarranted). Relevant here, the Human Ecology Action League reported that one in five people in the United States are adversely affected by exposure to synthetic fragrances in perfumes and cleaning products (Kosta, 1998). It is interesting to note how many of the scents that have been reported to improve our well-being are related to nature (e.g., plants and flowers; Warren and Warrenburg, 1993; Weber and Heuberger, 2008; Chen, 2009; Jo et al., 2013; Glass et al., 2014; Darabia and Mirabi, 2018). The latter observation, then, might link to evolutionary psychology, i.e., the biophilic account that has been put forward in order to help explain our attraction to green scenes of nature that incorporate water (cf. Wilson, 1984; Grinde and Patil, 2009; Steinwald et al., 2014; Gillis and Gatersleben, 2015; McMahan and Estes, 2015).

Researchers have shown that ambient urban odors are capable of evoking basic emotions in people of different ages (Weber and Heuberger, 2011; Glass et al., 2014; Glass and Heuberger, 2016). For instance, in one study, Glass et al. (2014) demonstrated that exposure to a range of typical ambient urban odors (synthetic mixtures representing the odors of disinfectant, candles/bees wax, summer air, burnt smell, vomit, and a musty smell) gave rise to significant changes in autonomic activity (e.g., in the electrodermal response). And, in one of the larger of the more recent studies of the impact of pleasant ambient fragrance, Haehner et al. (2017) conducted a single-blinded between-participants experimental study assessing the effect of short-term exposure to room fragrance on people’s attention, anxiety, and mood.

The 200 Germans who took part in the latter study spent around 30 min completing a number of standardized psychological tests (of attention, anxiety, and mood) in a room that had a grapefruit aroma, a combined lemon-lime-orange aroma, a nature-identical synthetic rose aroma, or else had been left unfragranced. The scents consisted of essential oils for the fruit aromas and nature-identical synthetic rose odor dispensed by a commercially available room-fragrancing device. The results revealed no main effect of room fragrance on any of the measures. That said, separating the results by sex revealed that the grapefruit scent appeared to have a slightly more positive effect on the male participants. Normally, it is women who tend to be more influenced by ambient odors than men (e.g., see Marchand and Arsenault, 2002). The latter showed something of a negative response (i.e., lower attention, higher anxiety, and impaired mood) to the rose-scented room. At the same time, however, it should be noted that the effects on mood, anxiety, and attention in men in this study were only marginally significant, and, what is more, failed to survive the correction for multiple comparisons.

Furthermore, the between-participants nature of Haehner et al.’s (2017) experimental design makes interpretation of the results of this particular study somewhat more challenging, because of the possibility of underlying between-groups differences. Unfortunately, the participants were not asked what they thought about the fragrance, nor even whether they had detected it (cf. Forster and Spence, 2018), nor how intense they found it to be. Moreover, no calculation was reported to assess whether the researchers in question had themselves collected a sufficiently large sample size in order to detect any significant between-group differences should they have been present. On the other hand, as Haehner et al. (2017) themselves noted, many of the earlier studies that have reported a significant effect of aromatherapy fragrance on people’s mood, well-being, and performance have tended to be statistically underpowered (by contemporary standards) and were often not blinded, hence raising a different set of concerns about the robustness of the results reported.

### Interim Summary

Taken together, the most parsimonious conclusion to draw on the basis of the literature that has been published to date is that while a significant (and typically positive) impact of ambient fragrancing on human well-being has often been reported, it is by no means guaranteed to occur. Furthermore, as yet, there isn’t a clear sense of what the key factors determining whether an effect of ambient scent will be demonstrated or not (see also Warren and Warrenburg, 1993; Goel and Grasso, 2004). Potentially relevant here is the fact that our response to an ambient fragrance is likely to depend on its intensity, whether we personally happen to like it, not to mention our perception, or belief, about it being synthetic/artificial versus natural (e.g., Herz, 2000; Wilkins et al., 2007; Baccarani et al., 2020). People’s belief about the efficacy of ambient scent, and its likely effect(s), also seems to play a role too (Torii et al., 1988; Lorig and Roberts, 1990; Campenni et al., 2004; Moss et al., 2006). This observation has led some researchers to suggest that the effect of pleasant ambient scents may be a kind of placebo effect. At the same time, however, it is clear that conditioned associations resulting from prior exposure also represent an important factor determining the effect of specific odorants (Robin et al., 1999). The duration of people’s exposure to ambient fragrance may also be relevant, varying between 30 min in Haehner et al.’s (2017) study (essentially reporting null results) and 2 h made up of 30 min exposure periods spread out over the course of a day in Sakamoto et al.’s (2005) study, where significant effects of scent were reported. And finally, it is perhaps also worth bearing in mind Warren and Warrenburg’s (1993, p. 12) earlier observation that: *“When a normal subject is at rest, the effects of odors on the peripheral nervous system appear to be minimal and difficult to measure.”*

## Home and Office Smells

Two of the locations in the built environment where we spend most of our time are the home and work, and it is to these that we will turn next. As will become clear in this section, the type of odorant that might be most appropriate in terms of enhancing our well-being depends on the particular circumstances and state of mind that we find ourselves in. What is more, a number of the odorants that we are exposed to on a regular basis are not necessarily related to ambient fragrances (such as air fresheners) but are perhaps better classified as incidental building odors. Baudelaire once described the smell of a room as ‘the soul of the apartment’ (Corbin, 1986, p. 169, and there is certainly no shortage of advice out there concerning the best scents to sell a (new) home; Demetros, 1997; Anon, 2014; Ebert, 2018). At the same time, however, the smell of cigarette smoke and pets, and here we are talking about dogs not goldfish, can be particularly off-putting, potentially reducing sale prices by as much as 10% according to the CEO of one Florida estate agency (McCooey, 2008; Anon, 2014). At the very upper end of the housing market, the lucky owners of one high-end apartment commissioned synesthetic scent-designer Dawn Goldsworthy to create a bespoke scent especially for the $29 million new Miami condo back in 2018. The idea was for this fragrance to be dispersed through the air conditioning, thus giving their property a truly unique olfactory identity (Schroeder, 2018).

At the same time, however, there is little doubting that specific incidental ambient odors associated with place can be incredibly evocative too, as anecdotally noted by Pallasmaa (1994, p. 32) when he wrote that: *“The strongest memory of a space is often its odor; I cannot remember the appearance of the door to my grandfather’s farm-house from my early childhood, but I do remember the resistance of its weight, the patina of its wood surface scarred by a half century of use, and I recall especially the scent of home that hit my face as an invisible wall behind the door.”* Once again, though, note how it is the personal associations that are formed with the smell, rather than the particular smell itself, that are key to understanding ambient smell’s emotional impact on someone (Reid et al., 2015; see also Herz, 2016, for a review).

Traditionally, the home would have been the place where the majority of us would have been exposed to food and cooking aromas. The basement kitchens that were such a common feature of Victorian-era homes in the United Kingdom released food cooking aromas throughout the house. Concerned by the regular exposure to cooking smells, the architect J. J. Stevenson wrote in 1888 that: *“unless the kitchen itself is ventilated so that all smells and vapors pass immediately away, they are sure to get into the house, greeting us with their sickly odor in the halls and passages, and finding their way to the topmost bedroom, notwithstanding all contrivances of swing doors and crooked passages”* (Stevenson, 1888). The famous Swiss architect Le Corbusier tackled this particular problem in the house he designed in Poissy, just outside Paris by deliberately placing the kitchen on the roof to avoid scents from the kitchen from permeating the rest of the house (Steel, 2008).

One of the most frequently studied effects of ambient fragrance is in terms of its alerting/relaxing function. There has been growing interest in the influence of fragrance on the performance of workers. In one Japanese car factory, for example, a significant increase in productivity (together with a concomitant reduction in accidents) was reported when a lemon scent was introduced onto the factory floor (see Shimuzu Corporation, 1988; Knasko, 1992; Barker et al., 2003; Zoladz and Raudenbush, 2005; Herz, 2007; Shepherd, 2010). Positive results of odor administration on people’s performance have not always been reported though (e.g., see Marx, 1990; Gaygen and Hedge, 2009). An increase in positive affect is one of the mechanisms by which ambient scent affects us (Baron and Thomley, 1994), though it is by no means the only one.

A number of researchers have argued that lavender can sometimes be used to enhance productivity by reducing stress (Ludvigson and Rottman, 1989; Motomura et al., 2001; Sakamoto et al., 2005). At the same time, however, lavender has long been mentioned in plays and literature as a scent that is commonly associated with sleep (Kirk-Smith, 2003). In fact, there is now a broad range of research demonstrating beneficial effect on sleep quality of exposure to ambient lavender scent across the lifespan (e.g., Burns et al., 2002; Field et al., 2008). That said, lavender has also been used to improve mood (Goel and Grasso, 2004). Here, though, it should be born in mind that there are at least 45 species and 450 varieties of lavender according to the United States Lavender Growers Association website, and often it is unclear which species of lavender has been used in many of the academic studies, nor is it clear how much of a difference this might make (Spence, 2003).

There is a sense in which different scents might be appropriate for different locations in the home. Given that we spend something like a third of our lives asleep (or at least trying to; see Walker, 2018), then those scents that can help us to sleep are potentially very important. At other times, an alerting scent may be more appropriate (Warm et al., 1991; Diego et al., 1998; Moss et al., 2003). In this regard, peppermint odor has often been shown to be an effective alerting smell (Warm et al., 1991; Ho and Spence, 2005; Moss et al., 2008). At the same time, however, it is worth noting that expectancy/belief regarding the efficacy of certain essential oils also appears to modulate the effect that they actually have on people (Moss et al., 2006).

As well as positively influencing our mood and emotion, it has been suggested that certain semantically meaningful scents are also capable of priming specific behaviors (e.g., Baron, 1990). So, for example, according to the results of a couple of published studies, people engage in significantly more cleaning, and are more likely to pick up rubbish, with a citrus cleaning scent in the air (Holland et al., 2005; De Lange et al., 2012; though see also Toet et al., 2013, for evidence suggesting that scents may have somewhat different effects in virtual environments). Meanwhile, other researchers have reported that the presence of ‘clean’ ambient scents (a spray of citrus-scented Windex) can also promote reciprocity (in a one-shot anonymous trust game) and charitable behavior (e.g., as assessed by the intention to volunteer; Liljenquist et al., 2010). At the same time, however, the robustness of a number of these smell-induced behavioral priming effects have also been questioned by researchers (Smeets and Dijksterhuis, 2014; see also Doyen et al., 2012).

By now, a wide variety of largely aromatherapy odorants have been reported to have beneficial effects. It remains somewhat uncertain though as to whether the beneficial effects of aromatherapy scents can be explained by priming effects, based on associative learning, as in the case of the clean citrus scents mentioned above (see Herz, 2009), versus via a more direct (i.e., less cognitively mediated) physiological route (cf. Harada et al., 2018). While the associative learning account of ambient odor’s influence on us undoubtedly explains much of the data, there is nevertheless some evidence to suggest that certain odors may have a more direct physiological effect (Saeki and Shiohara, 2001).

While the scent of air fresheners and cleaning products have been reported to have a positive effect on various aspects of behavior, mood, and well-being (Hosey, 2013), it is worth remembering that the airborne chemicals, and/or the result of their reaction with other contaminants that may be present in the indoor environment, may themselves constitute a form of air pollution (see Nazaroff and Weschler, 2004, for a review of the adverse health consequences associated with inhaling the chemicals that can be found in cleaning products; and Senger, 2011, on the sensitivity to perfume scent). It is important to note that while the building odor of our own home is one of the ambient smells that we are likely to be exposed to most frequently, it is also one of the smells that we are least likely to be aware of (see Dalton and Wysocki, 1996; McCooey, 2008; see also Našel et al., 1994).

## Signature Scents: Leading the Consumer by the Nose

In recent years, there has been growing awareness of the marketing effectiveness of various pleasant scents, especially food scents, thus perhaps helping to explain why it is that so many of us are now exposed to more food aromas in the built environment than perhaps ever before (see Spence, 2015, for a review). In the context of the shopping mall, it has even been suggested that the owners of food chains such as Cinnabon etc, deliberately search out locations for their stores that are close to the bottom of enclosed stairwells, thus ensuring that their appealing smells reach more of their potential customers’ noses (see Nassauer, 2014, for a review). At the same time, however, a number of other traditional odorless environments have, in recent times, also acquired a food scent. Just think, for example, about how public spaces, such as book stores and train stations, now have the seemingly ubiquitous smell of coffee (Luttinger and Dicum, 2006, p. 164). Even clothing stores such as Uniqlo and Club Monaco have been getting in on the act (see Oldenburg, 1989, p. 39; Cheng, 2014).

There have even been reports of Japanese building firms deliberately releasing food scents through the air-conditioning of the building at different times on different floors, in order to help manage the flow of people to the restricted capacity canteen (Spence, 2002; Herz, 2007). Given the evidence showing that food smells influence our food choices (Gaillet-Torrent et al., 2013, 2014), one might therefore worry about whether we are all being ‘nudged’ by deliberate ‘scent-sory’ marketing strategies into eating more than we otherwise might (White, 2011; Spence, 2015)? The ubiquity of ambient food smells, and the unhealthy impact that such smells might be having on our waistlines, have led some commentators to suggest that many of us should perhaps be wearing inserts in our nostrils to help reduce our exposure to ambient food smells, and thus help fight olfactory temptation^3^ (e.g., Best, 2018; though see also Biswas and Szocs, 2019).

There has long been awareness of the marketing potential associated with various non-food smells (Mitchell et al., 1964; Miller, 1991, 1993; Hunter, 1995; Goldkuhl and Styvén, 2007), and commercial interest in scent marketing would appear to be on the rise (Trivedi, 2006). In the best case scenario, an ambient scent is deliberately introduced that happens to be both distinctive, so that it can be recognized by the consumer, and hence be associated with a specific brand (this is what is known as a signature scent; Minsky et al., 2018). At the same time, however, it should also deliver a functional benefit as well – be that in terms of improving the customers’ mood or increasing sales (see Spence et al., 2014, for a review). And while much of the research in scent marketing thus far has been led by those chains whose products naturally smell,^4^ the approach has, in recent years, been extended to a number of other product categories, where the products themselves do not necessarily have their own distinctive scent (think here only of white goods and consumer electronics; e.g., Trivedi, 2006).

At the present time, there is a large literature demonstrating the impact of scent on various aspects of shopper behavior (e.g., Gulas and Bloch, 1995; Spangenberg et al., 1996, 2006; Fiore et al., 2000; Chebat and Michon, 2003; Davies et al., 2003; Milotic, 2003; Ward et al., 2007; Orth and Bourrain, 2008; Bradford and Desrochers, 2009; Morrison et al., 2011; Vinitzky and Mazursky, 2011; Teller and Dennis, 2012; Doucé et al., 2013; Madzharov et al., 2015; Rimkute et al., 2016; see Gilbert, 2008, Chapter 9: “Zombies at the mall,” for a review). Although there is simply not the space to review all of this research here, it should be recognized that there are likely to be a range of different mechanisms at play in terms of helping to explain the effects of the presence of an appealing/pleasant ambient scent on consumers in commercial and public spaces. On the one hand, ambient smells can influence people’s approach-avoidance behavior (e.g., Pacelle, 1992). Relevant here, it has been reported that consumers find simpler scents easier to process than they do more complex scents, and that this may exert an influence over their approach behavior (Herrmann et al., 2013). Though note that defining what, exactly, is meant by ‘complex’ in this context, is by no means a simple matter to ascertain (see Spence and Wang, 2018).

As we have seen already, ambient scent sometimes influences people’s mood and arousal as well (Leenders et al., 2019), and can apparently even enhance pro-social behaviors (Baron, 1997a,b) not to mention social interaction (Zemke and Shoemaker, 2007, 2008). Thereafter, semantic associations with scent, including with signature scents (Danziger, 2017; Minsky et al., 2018), may become important, priming certain thoughts, associations, and even behaviors (i.e., think of it as a kind of olfactory branding effect). Finally, there are also the more-or-less idiosyncratic individual associations that many of us form with specific scents (Pallasmaa, 1994; Van Campen, 2014; Herz, 2016). There are, in other words, multiple routes to the impact of ambient smells on consumer behavior and well-being. At the same time, however, one also needs to consider the ethical implications of ambient scenting (see also Hirsch, 1995, for one particularly worrying example; Spence, 2020c). Ultimately, though, it can be argued that only limited progress will be made in understanding the impact of ambient scent on well-being, or anything else (e.g., store sales), by considering the sense of smell in isolation from the other senses.

## Smell in the Multisensory Built Environment

Our well-being (not to mention our behavior) is determined not just by the olfactory ambience, but rather by our response to the total multisensory environment (Spence et al., 2014). As such, it can be argued that it makes little sense to focus solely on ambient scent when considering the impact of the sensory aspects of the built environment on well-being. After all, the senses presumably interact to determine our perception of the environment/atmosphere (Böhme, 2019), just as they do in many other aspects of our everyday perception (Spence, 2020e, 2021). Architectural theorists are increasingly coming to recognize this. Just take Malnar (2017, p. 146), who has noted that: *“The point of immersing people within an environment is to activate the full range of the senses.”* Meanwhile, some years earlier, Pallasmaa (2000, p. 78) made a similar point, writing that: *“Every significant experience of architecture is multi-sensory; qualities of matter, space and scale are measured by the eye, ear, nose, skin, tongue, skeleton and muscle.”* (see also Feld, 1996, p. 99).

The problem, though, is that, as yet, there has been relatively little research directed at the question of how atmospheric/environmental multisensory cues actually interact to influence our perception, behavior, mood, and well-being (Verissimo and Pereira, 2013; see also Velasco et al., 2014). Mattila and Wirtz (2001, pp. 273–274) drew attention to this lacuna two decades ago, when they wrote that: *“Past studies have examined the effects of individual pleasant stimuli such as music, color or scent on consumer behavior, but have failed to examine how these stimuli might interact.”* To date, only a relatively small number of studies have directly studied the influence of combined ambient/atmospheric cues on people’s perception, feelings, and/or behavior, with much of this multisensory research having been conducted in the field of sensory marketing, which is where we turn first.

In one influential early study, Mattila and Wirtz (2001) manipulated the olfactory environment (no scent, a low arousal scent [lavender], or a high arousal scent [grapefruit]) while simultaneously manipulating the presence of music (no music, low arousal slow-tempo music, or high arousal fast tempo music) in a physical retail outlet. Intriguingly, when the scent and music were congruent in terms of their arousal potential, the customers (or, at least the 270 of them who agreed to be interviewed) rated the store environment more positively, exhibited higher levels of approach and impulse buying behavior, and expressed more satisfaction.^5^ It is unclear on the basis of Mattila and Wirtz’s results whether the effects of a novel combination of grapefruit scent and high-tempo music (which was the congruent combination of stimuli that appeared to be driving the effects reported) might soon wear off. As yet, I am not aware of anyone having conducted the appropriate studies to investigate how long-lasting the influence of such atmospheric multisensory cue combinations might be in the marketplace (though see Girard et al., 2019a,b, for a preliminary start in this direction).

A few years later, Spangenberg et al. (2005) conducted a study in which they attempted to assess the consequences of combining congruent Christmas-related scent (the mysterious-sounding ‘Enchanted Christmas’) and music on students’ evaluation of a department store, as seen in a series of photographs showing a store and its merchandise. A total of 140 students responded regarding their attitude toward the store, its merchandise, and their likelihood of visiting. On the basis of their results, Spangenberg et al. (2005, p. 1588) concluded that: *“Retailers need to be aware that not all combinations of music and scent positively affect shoppers. Non-congruent combinations are unlikely to elicit favorable outcomes. Retailers might be better advised to use a single environmental cue rather than incongruent combinations of music and scent.”* In fact, one might be tempted to go even further, given that nothing in Spangenberg et al.’s (2005) results actually suggested that congruent olfactory and auditory cues were any better than a well-chosen auditory track by itself. That is, the highest store ratings were documented when the non-Christmas music was presented in the absence of any specific ambient scent. At the same time, however, it is worth bearing in mind here, given that Spangenberg et al. (2005) only presented a series of pictures to the participants, other findings suggesting that scents do not have quite the same effect in virtual reality environments (Toet et al., 2013; see also Jiang et al., 2016).

In another study, conducted by Morrin and Chebat (2005), adding scent and sound in the setting of a North American Mall reduced unplanned purchases as compared to either of the unisensory interventions amongst almost 800 shoppers. Although they provide no evidence to support their claim, Morrin and Chebat (2005, p. 188) suggested that the suppression of unplanned sales in their multisensory condition might have reflected the consequences of sensory overload (Malhotra, 1984). However, an alternative possibility is that the combination of low-tempo relaxing music (60 beats per minute) with a complex citrus scent (comprising three citrus notes, that was likely alerting) may have been perceived by shoppers as incongruent, and hence lacking in processing fluency (Reber, 2012; Herrmann et al., 2013; Winkielman et al., 2015).

Fenko and Loock (2014) conducted an intriguing study in a plastic surgeon’s office in Recklinghausen, Germany (*N* = 117 participants in a between-participants design). While the addition of the pleasant and relaxing scent of lavender or pleasant music with nature sounds (both stimuli were chosen to be high on pleasantness and low on arousal) helped to reduce anxiety for those patients waiting for their appointment, combining these two cues was actually found to be no more effective that the ‘no stimulation’ baseline condition. What is more, the sensory interventions had no effect on perceived waiting time either. While the stimuli were chosen to be congruent in terms of their pleasantness, it is unclear whether they were also perceived to be semantically congruent or not. As such, these results highlight the need to consider olfactory interventions in the context of the total multisensory atmosphere.

Recently, Ba and Kang (2019a) examined the nature of any crossmodal interactions between ambient sound and smell in a laboratory study designed to capture the sensory cues that might be encountered in a typical urban environment. In particular, these researchers paired the sounds of birds, conversation, and traffic, with the smells of flowers (lilac, osmanthus), coffee, or bread, at one of three levels (low, medium, or high) in each modality giving rise to a total of 129 stimulus combinations (consisting of both unimodal and multisensory stimulus presentations). The stimuli were rated by 168 student participants in terms of their comfort, liking, familiarity, intensity, and, for the auditory-olfactory stimulus combinations, their crossmodal congruency. Perhaps unsurprisingly, a complex array of interactions were documented, with increasing stimulus intensity sometimes enhancing the participants’ comfort ratings, while sometimes leading to a negative response instead.

Although the very large number of stimulus conditions (*N* = 129) that were assessed in their study makes the drawing of any simple conclusions difficult, taken together, the complex array of significant findings reported nevertheless clearly do suggest that sound and scent can interact in terms of influencing people’s evaluation of urban design (see also Ba and Kang, 2019b). Ba and Kang (2019a, p. 314) concluded that: *“For the overall evaluation, in the presence of birdsong and low-volume sound, the overall evaluation was unaffected by odor; for other combinations of sound and odor, with increased concentration, the overall evaluation improved*…. *a positive sensory stimulus can improve the evaluation of other senses, while a negative one has the opposite effect. There is a masking effect between audition and olfaction that is reflected in the finding that when one stimulus is stronger, the other has weaker perceptual intensity;*… *Furthermore, the relationship between sensory evaluation and overall evaluation showed that for overall comfort, the effect of sound was stronger than odor.”*

### Interim Summary

As the various results reported in this section make clear, it is the multisensory atmosphere that is ultimately going to determine how the smell of the built environment affects our well-being (Spence, 2002, 2020e, 2021). As the limited results that have been published to date in this area illustrate, combining olfactory and auditory cues (as but one example of combining multisensory atmospheric cues), does not necessarily give rise to a positive outcome in terms of either people’s well-being or product sales (Mattila and Wirtz, 2001; Morrin and Chebat, 2005; Spangenberg et al., 2005; Fenko and Loock, 2014; Ba and Kang, 2019a). Two of the key issues when thinking about smell as one component of the multisensory built environment are the dangers of sensory overload on the one hand (Malhotra, 1984) and sensory incongruency on the other. The latter, note, typically leading to a negatively valenced reduction of processing fluency (e.g., Reber, 2012; Winkielman et al., 2015). Note that similar challenges likely arise when considering the multisensory combination of visual, olfactory, and possibly also auditory cues (Pelgrim et al., 2006; Pavia, 2008; Jiang et al., 2016).

While congruency is often mentioned as a relevant construct in multisensory research, it is important to remember that it can be defined at multiple levels. For instance, as we have seen already in this section, sensory cues (such as scent and sound) may be more or less congruent in terms of their arousal/relaxation potential (e.g., Mattila and Wirtz, 2001), or in terms of their pleasantness (Fenko and Loock, 2014). At the same time, however, ambient sensory cues may also be rated as more or less congruent in terms of some more abstract crossmodal correspondence (Spence, 2020b) or semantic associations (Dalton et al., 2008), which can themselves operate at different levels of abstraction (Spangenberg et al., 2005). Ultimately, it is not altogether clear whether we maintain any particular priors about whether the various environmental cues should be congruent in the way that, for example, we have been shown to do when perceiving the various unisensory cues associated with a particular object or event (see Chen and Spence, 2017, for a review on the latter).

## Conclusion

Although typically neglected, given our visually dominant nature (see Hutmacher, 2019, for a review), there is a growing realization that the smells that are associated with our predominantly indoor existence may be having a significant impact on our mood and well-being (see Spence, 2002, 2021, for reviews). This goes together with an emerging realization that the sense of smell is more important to human well-being than has been suggested by many scientists over the last century (McGann, 2017). Oftentimes, the focus with research on ambient smell’s influence on well-being tends to be on the negative impact when considering phenomena such as SBS (Redlich et al., 1997; Love, 2018). At the same time, however, the emerging evidence also highlights the potentially positive impact that pleasant ambient scents can have on our social, emotional, and cognitive well-being, at least if managed appropriately. Scents, very often those associated with flowers, herbs, and spices, and other plants can be used to help us relax and sleep better at night, while making us more alert and productive during the day (see Spence, 2021, for a review). At the same time, however, it is worth reiterating Warren and Warrenburg’s (1993) cautionary note that fragrance-evoked mood changes tend to be small, and that the stress-reducing response of fragrance in humans may be difficult to measure in those individuals who are not stressed to begin with.

In 1990, Shiseido Co., Ltd. of Tokyo (a leading manufacturer of cosmetics) reported, as part of their ‘aroma engineering program’ that releasing the right scent in the factory led to increased productivity and reduced accidents when pumped through the air-conditioning and heating ducts (see Warren and Riach, 2018). According to Gary Marx: *“The company’s promotional brochure suggests one menu: lemon scent in the morning to wake workers up; a light floral scent to aid concentration at mid-morning; an odorfree lunch, and wood, lemon and floral scents in the afternoon.”* Rather than thinking that one scent will necessarily suffice to improve well-being regardless of the situation, there is a very real sense in which different smells may be needed at different times (and in different places; e.g., see Gorman, 2017). Of course, such a dynamic approach to scenting the built environment likely requires progress to be made in terms of scent-release technology. That said, many of the home scent-delivery systems capable of delivering a range of odorants have not fared very well: think here only of Digiscents (e.g., Dusi, 2014), or P&G’s ill-fated ‘scent-stories’ (see Herz, 2007). And while acknowledging the beneficial effects on mood and relief from stress that certain pleasant ambient fragrances may deliver, one also needs to be sensitive to the olfactory pollution that the fragrance of various cleaning and household fragranced products might give rise to (e.g., Lau et al., 1997; Anderson and Anderson, 1998; Nazaroff and Weschler, 2004; Caress and Steinemann, 2009; De Vader, 2010; Petry et al., 2014; Steinemann, 2016, 2019; Pain, 2017; Sarchet, 2017; Scully, 2019).

### Ambient Smell and Evolutionary Psychology

As to why the right balance of olfactory stimulation should influence us, one might think in terms of evolutionary psychology (Wilson, 1984). It has been suggested that we may have evolved to appreciate those sights and sounds, and even those natural smells that would have helped our ancestors to survive. Given that our preference as far as the thermal conditions of the home environment has recently been shown to match most closely the temperature and humidity profile of the Ethiopian highlands where we evolved some five million years ago (see Just et al., 2019; Whipple, 2019), one might, I suppose, wonder whether we have also evolved to find pleasant those scents that would have been familiar/present in the environment at that time? That said, one of the interesting features about smell is the fact that we rapidly learn to associate meaning/mood/emotion with novel scents (e.g., Engen, 1982; Herz, 2007). It is certainly unlikely that many of the specific plant odors that have been demonstrated to affect us nowadays would have necessarily been present in Ethiopia all those many years ago.^6^

The other part of the evolutionary olfactory story is presumably the world smells more strongly during the warmer part of the day, and typically smells less fragrant at night (with the rare exception of plants such as night-flowering jasmine, Spence, 2021). As such, one might expect that our sensitivity to scent to be adapted to that natural cycle or rhythm (cf. Smith, 1977; Knowles, 2006). By contrast, the built environment would seem to have a much more constant level of olfactory stimulation – or at least it does in those parts of the built environment that are inhabited at night. However, the limited evidence that has been published to date has yet to provide support for this particular suggestion, appealing though it might be (Lötsch et al., 1997; Carskadon et al., 2015; though see also Goel and Grasso, 2004).

While the research that has been reviewed in this admittedly Anglophone article has focused almost exclusively on those smells that have been experienced indoors in western urban environments, it is worth noting that a parallel literature exists documenting the impact of the outdoor scents of the urban environment on us. In fact, there is growing interest in urban scentscapes (e.g., Porteous, 1985; Margolies, 2006; Diaconu, 2011; Henshaw, 2014), as highlighted by those making scentscape maps of cities (see Reinarz, 2014; Reynolds, 2014; Lipps, 2018; McLean, 2018; see also Dann and Jacobsen, 2003). The range of scents that are typically experienced outdoors are likely capable of exerting both a positive and negative effect on our well-being, depending on their specific identity, and our beliefs concerning their origins (Day, 2007; Gorman, 2017). Separately, there is growing interest in the scenting of public transport, such as, for example, trains and airports (e.g., Haehner et al., 2017; Canniford et al., 2018; Girard et al., 2019a,b).

### Multisensory Atmospherics

The other important part of the equation when considering the smellscapes of the built environment (Xiao et al., 2018) is to consider the multisensory atmosphere. As the cultural geographer Porteous (1985, pp. 359–360) noted some years ago, odors make little sense without reference to the other senses. Mattila and Wirtz (2001, p. 285) made a similar point when they stated that it is *“the total configuration of cues that influence consumer responses”* rather than the atmospheric variables when considered in isolation. The relevant question here is whether or not the scent is congruent with the visual, and auditory aspects of the environment (Jiang et al., 2016; Ba and Kang, 2019a,b). The multisensory perception literature that has emerged over the last four decades or so suggests that when multiple cues are present, they may be integrated, revealing a pattern of sensory (normally visual) dominance, superadditivity, or, when the inputs are incongruent, sub-additivity.

Normally, we find it easier to process congruent combinations of multisensory stimuli – they tend to be processed more fluently (Reber et al., 1998, 2004; Reber, 2012). It would seem likely the same is true when it comes to the elements of the built environment, and hence we are presumably more likely to be drawn toward multisensorially congruent environments (Knasko, 1995; Spangenberg et al., 2005; though see also Mitchell et al., 1995; Schifferstein and Blok, 2002; Bosmans, 2006). At the same time, however, visual dominance means that the way we interpret a smell can sometimes be influenced by what we see (and potentially also hear around us; Ba and Kang, 2019a). And, as Rimkute et al. (2016) note, ensuring congruency can be difficult in non-uniform environments (i.e., in those stores offering multiple different classes of product).

While we appear to have a predisposition to integrate those sensory cues that belong to the same object or event (see Chen and Spence, 2017, for a review), it is, as yet, less clear whether we automatically integrate ambient atmospheric cues in quite the same manner. Certainly, it is less clear that people attend to atmospheric cues in quite the same way that they do when presented with objects or events in the laboratory (Spence, 2020a,e). At the same time, however, there is also a sense in which we may be conditioned to treat the various environmental cues as operating independently. Just think about how the music changes while the lighting and ambient scent stay the same in a restaurant, or when the lighting is turned down at some point in the evening, without there being any concomitant changes to the other sensory aspects of the environment. There is, though, a very real danger that by trying to study the effect of the multisensory built-environment on well-being, researchers may end up drawing their participants’ attention to, and hence making more salient, the relative (in-)congruency of the component ambient stimuli presented in a way that would perhaps not normally be the case.

Looking to the future, especially during the era of pandemia, what is needed are more long-term studies (Girard et al., 2019a,b), and more studies conducted in relevant spaces in the built environment, be they offices, shops, home or transport, rather than just more laboratory studies with correspondingly low ecological validity. At the same time, given the multisensory nature of the built environment, it will be important to learn more concerning the various ways in which ambient smell interacts with other environmental sensory cues to determine how the built environment affects our well-being.

## Author Contributions

CS wrote all parts of this review.

## Conflict of Interest

The authors declare that the research was conducted in the absence of any commercial or financial relationships that could be construed as a potential conflict of interest.

## Footnotes

1 This referencing Brian O’Doherty’s (1999, 2009) earlier discussion of the ‘white cube’ mentality in art galleries.

2 Of course, associative learning is likely also responsible for certain odors being rated as smelling sweet in the first place (Stevenson and Boakes, 2004).

3 https://noznoz.com/

4 Just think about how the distinctive smell of Lush cosmetics can often be detected many meters down the street. In this case the company does not package many of their products, hence meaning that it is the authentic smell of their products that is being detailed in this case.

5 Of course, given that only one example or a low arousal and a high arousal scent was used in Mattila and Wirtz’s (2001) study, it is impossible to know whether it is the how arousing the scent was, or its specific sensory qualities, that were key to driving the effects reported in this study.

6 Though I suppose it might explain our strong response to geosmin, the distinctive smell of the earth after it has rained (Pelosi, 2016).
